# Hemostatic Efficiency and Wound Healing Properties of Natural Zeolite Granules in a Lethal Rabbit Model of Complex Groin Injury

**DOI:** 10.3390/ma5122586

**Published:** 2012-12-03

**Authors:** Yunlong Li, Hui Li, Liping Xiao, Lin Zhou, Jianzhong Shentu, Xumin Zhang, Jie Fan

**Affiliations:** 1 Key Lab of Applied Chemistry of Zhejiang Province, Department of Chemistry, Zhejiang University, Hangzhou 310027, China; E-Mails: liyunlong@zju.edu.cn (Y.L.); lpxiao@zju.edu.cn (L.X.); 2 Key Lab of Combined Multi-Organ Transplantation, Ministry of Public Health, Department of Hepatobiliary and Pancreatic Surgery, First Affiliated Hospital, School of Medicine, Zhejiang University, Hangzhou 310003, China; E-Mails: felixcat111@163.com (H.L.); linzhou19@163.com (L.Z.); stjzcn@yahoo.com.cn (J.S.); 3 Zeo-Innov Medical Technology Corporation, Hangzhou 310023, China; E-Mail: zxm@hzkm.cn

**Keywords:** natural zeolite, hemostatic efficiency, mortality, wound healing, groin injury

## Abstract

Quikclot has been used many years for treating external hemorrhage in the battle field. In this study, the hemostatic performance of NZG-JY (natural zeolite granules from Jinyun, China) was evaluated and compared with Quikclot in a lethal rabbit model of complex groin injury. Fifty-six anesthetized rabbits were randomized to three different groups: (1) NZG-JY (*n* = 19); (2) Quikclot (*n* = 19); and (3) medical gauze (*n* = 18). Survival was recorded three hours after the application of the hemostatic agents. The wound healing properties of the survived animals (*n* = 4 for each group) were observed a week later. The clotting efficiency is 100% for the animals in the NZG-JY and the Quikclot group, while only 5.6% in the gauze group. The mortality in the NZG-JY group (21.0%) is significantly less than that in the Quikclot group (52.6%) and the gauze group (66.7%). A good healing property was achieved in all animals that survived in the NZG-JY group, while three quarters of the animals in the Quikclot group had serious necrotic tissue. NZG-JY significantly decreases the mortality in a lethal rabbit model of complex groin injury and demonstrates good healing properties.

## 1. Introduction

Severe blood loss due to arterial bleeding is life threatening for the wounded in battlefields, civilian victims of vehicle accidents, street violence, wilderness accidents and construction incidents. Rapid hemostasis is crucial not only for decreasing mortality in these conditions, but also for optimal recovery [1].

In the last decade, many hemostatic agents have been proposed to facilitate hemostasis in severe bleeding [2,3,4,5,6,7,8]. The ideal hemostatic agent would be easy to use, highly effective, biocompatible, durable and inexpensive. One of the most attractive hemostatic agents is Quikclot, which had been used for many years by the U.S. forces in Iraq and Afghanistan for stabilizing life-threatening injuries [8,9,10]. There are still other inorganic hemostatics approved by the U.S. Food and Drug Administration, such as clay-based hemostatic Woundstat, which also show high survival in lethal extremity arterial hemorrhage [11]. However, significant endothelial and transmural damage was observed in wound treated vessels with thrombi and embedded Woundstat residues [12]. The U.S. Army halted the use of Woundstat powder designed for bleeding control only months after approving it. There are no such concerns for zeolite-based granular materials, such as Quikclot, which is one of the most popular hemostatic agents on the market. Quikclot can be externally applied directly to any wound in the field, by individuals with minimal medical training, to improve survival and reduce the complications of early blood loss.

**Figure 1:**
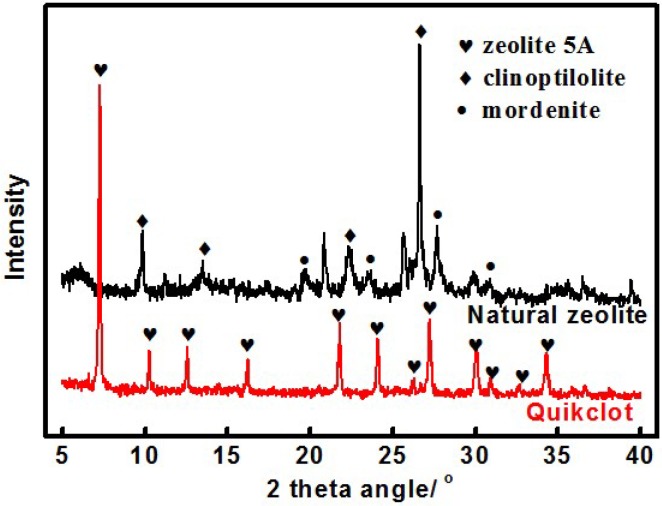
Powder X-ray diffraction pattern of Quikclot and NZG-JY (natural zeolite granules from Jinyun, China). Heart shapes identify reflections associated with the zeolite LTA-5A in Quikclot. Rhombus shapes indicate the high content of clinoptilolite in NZG-JY, and circular shapes indicate mordenite.

**Figure 2:**
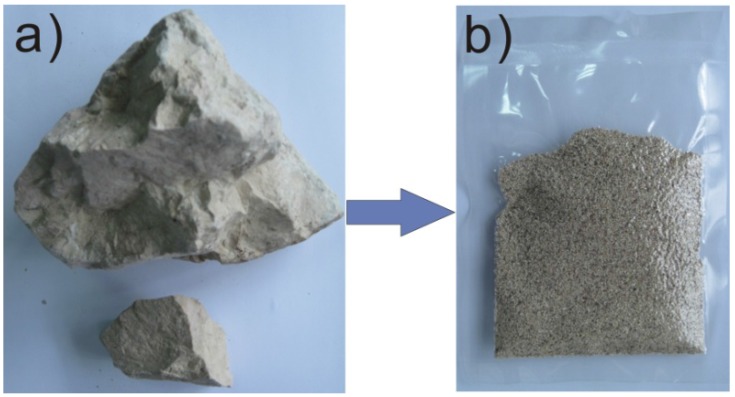
(**a**) The natural zeolite mineral from Jinyun, Zhejiang province, China; (**b**) The packed natural zeolite granules (manufactured by Zeo-Innov Medical Technology Corporation, Hangzhou, Zhejiang province, China).

The main component of Quikclot is a synthetic zeolite 5A with LTA structure (as show in Figure 1) [13], which does not exist in nature. However, natural zeolites hold some key advantages over their synthetic analogs. First, the manufacturing cost of natural zeolites is much less than synthetic zeolites (1/100), since they are abundant on earth. Second, the crystal size of natural zeolites is large enough for further manufacturing processes (e.g., granulation, Figure 2), which is impossible for synthetic zeolites. Finally, the non-biological properties and long-term physical–chemical stability in biological environments of natural zeolites make them excellent candidates for bio-medical application, such as diet additives, vaccine adjuvants, antibacterial agents, anticancer therapy and ion-exchange in hemodialysis processes [14,15,16,17,18].

The lethal animal model is widely used to evaluate *in vivo* hemostatic efficiency and to develop possible new blood clotting materials. Most previous research focused on animal models with arterial injuries only [3,11,19,20], which do not fully replicate battlefield or complex traumatic soft tissue or venous injuries that are associated with arterial injuries [8,10]. More importantly, rapid hemostasis is essential as a strategy, not only for initial survival, but also for optimal recovery. So far, there is no report on the wound healing properties of the animals that survived. In our work, NZG-JY not only significantly decreases the mortality in a lethal rabbit model of complex groin injury, but also demonstrates good healing properties.

## 2. Experimental Section

The objective of our study is to evaluate the hemostatic efficiency and wound healing property of natural zeolite granules (natural zeolite granules from Jinyun, China, denoted as NZG-JY, Figure 2b) in a lethal rabbit model of complex groin injury using Quikclot and medical gauze as the reference. The X-ray diffraction indicates that NZG-JY is the mixture of clinoptilolite and mordenite, in which the main component is clinoptilolite (Figure 1). NZG-JY, with sizes between 0.15 and 0.60 mm, was manufactured by Zeo-Innov Medical Technology Corporation (Hangzhou, China). Quikclot was purchased from Z-Medica.

### 2.1. Model and Animal Preparation

The study was performed by the Key Lab of Combined Multi-organ Transplantation, Ministry of Public Health, Department of Hepatobiliary and Pancreatic Surgery, First Affiliated Hospital, School of Medicine, Zhejiang University. All research was conducted in compliance with the Animal Welfare Act. The study was approved by the Zhejiang Medical Animal Care and Use Committee and adhered to the Regulations for the Administration of Affairs Concerning Experimental Animals.

Rabbits weighing between 1.5 and 2.5 kg were used as the participants for the animal experiments. The rabbits were bought from Zhejiang Academic of Medical Science and kept in cages for three days to ensure a good state of health. The animals were fed a standard diet with free access to water prior to the experiment.

**Figure 3:**
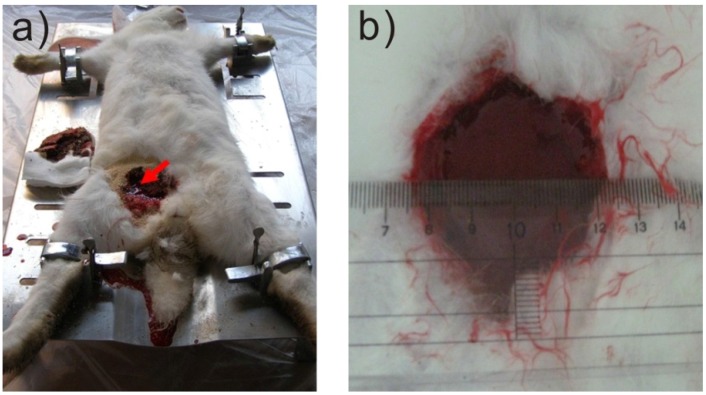
The lethal rabbit model with complex groin injury. (**a**) The wound applied with the Quikclot. Hemostasis was achieved after 3 min oppression; (**b**) The Wound applied with medical gauze, blood is oozing after removing medical gauze. The wound is around 4 cm long and 1 cm deep.

The hemostatic efficiency and wound healing properties were evaluated and compared in a lethal rabbit model of complex groin injury. The environment temperature was regulated between 25 °C and 35 °C. The rabbit was first fixed on the operating table and anesthetized with Ethyl carbamate (20%) by 3.5 mL/kg with mainline (Figure 3). A complex groin injury was created in 56 rabbits to produce an uncontrolled hemorrhage. This injury included transection of the proximal thigh soft tissue, complete division of the left groin artery, vein, muscle and nerve just below the groin. This was achieved by cutting these structures with medical scissors. An exposure and cut about 1 cm deep and 4 cm long in the groin was performed to produce the injury. All animals were randomized to be treated with either the medical gauze (*n* = 18), 12–15 g of NZG-JY (*n* = 19) and 12–15 g of Quikclot granules (*n* = 19), immediately. NZG-JY and Quikclot granules were directly poured into the wound over the vascular injury site, covered with three pieces of gauze, followed by application of direct pressure with the hands. Five piece of gauze were used when medical gauze was used alone. The gauze is about 8 cm × 10 cm in size and has eight layers. Figure 3 demonstrates the Quikclot granules and medical gauze applied to a severe groin injury.

In all groups, the compression was maintained for 3 min. After that, the gauze was carefully removed and the hemostasis was evaluated. No additional treatment would be applied, even if the resumption of bleeding was observed. The hemostasis was judged to be complete if there was no oozing of any kind after 20 s of visual observation by two separate laboratory assistants. Mortality was monitored for 3 h after the cut was produced. If the animals began to hemorrhage from the wound site during this course, no additional treatment would be applied. After 3 h, if the animals were still alive, the incision was cleaned with physiological saline and sterilized with hydrogen peroxide solution. Finally, the wound was closed with stitches. All the rabbits lost use of their legs after the experiment. The wound healing condition of the surviving animals (four rabbits for each group) was evaluated one week after survival. During this period, the animals were feed a standard diet with free access to water.

### 2.2. Measurement of Exothermic Reaction

The exothermic reaction of the zeolite-based hemostatic agents was studied *in vitro* by measuring the temperature increase of water after mixing with the hemostatic agents. In a typical experiment, 10 g of NZG-JY and Quikclot were separately added to a plastic tube (50 mL) inserted with a mercury thermometer, then 10 mL DI water was added into the tube, immediately. The highest reading of the thermometer was recorded.

### 2.3. Statistical Analysis

All data for the animal experiments are presented as group means ± SEM (standard error of the mean). The SPSS software program was used for inter-group comparisons. One-way analysis of variance (ANOVA) was used to compare animal weight. Unless specified otherwise, the medical gauze group was used as the control for statistical analysis. We used the *x*^2^ test to compare mortality rates against medical gauze and Quikclot groups. Significance was defined as *p* < 0.05. The data for exothermic reaction is also presented as means ± SEM.

## 3. Results and Discussion

The animal weight of each group is 1.89 ± 0.07 kg for the Quikclot group, 1.90 ± 0.07 kg for the NZG-JY group and 1.93 ± 0.08 kg for the medical gauze group; the animal weight does not display significant difference between groups (*p* = 0.924).

### 3.1. Mortality

There are significant differences for the hemostatic efficiency of the three applications in the animal experiment. Only 5.6% of the animals achieve hemostasis after gauze treatment, while all animals in the Quikclot and the NZG-JY groups achieve hemostasis. The complex groin injury causes 66.7% mortality in the control (medical gauze) group. The application of Quikclot granules decreases the mortality to 52.6%. However, there is no significant difference between the medical gauze group and Quikclot group (*p* = 0.385). The addition of NZG-JY offers a statistically significant (*p* = 0.005 *vs.* medical gauze group, *p* = 0.044 *vs.* Quikclot group) advantage by decreasing the mortality rate to 21.0%. The mortality rates in different experimental groups are listed in Table 1. These results demonstrate that the NZG-JY product is significantly better than Quikclot granules in decreasing the mortality in the lethal rabbit model.

**Table 1:** Post-injury clotting percent, mortality and wound infection differences of the three groups.

	Clotting percent	Surviving animals	Mortality	Wound infection	Infection percent
Quikclot	100%	9/19	52.6%	3/4	75%
NZG-JY	100%	15/19	21.0%	0/4	0
Medical Gauze	5.6%	6/18	66.7%	–	–

### 3.2. Wound Healing

**Figure 4:**
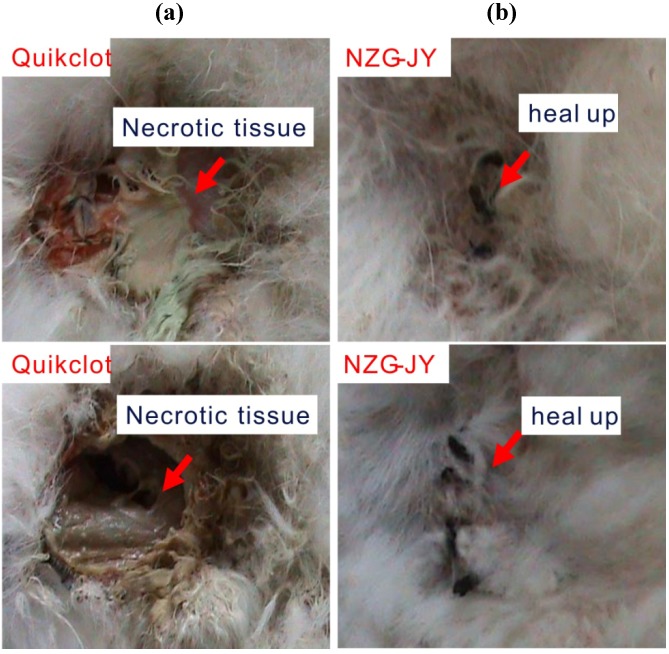
The wound healing condition of the surviving rabbits in the Quikclot group (**a**) and NZG-JY group (**b**), which was evaluated one week after survival. The rabbits in the Quikclot group have severe necrotic tissue; the rabbits in the NZG-JY group heal up well.

The wound healing properties of the surviving animals after NZG-JY and Quikclot treatments were observed and compared seven days post-wounding. The classic model of wound healing is divided into three or four sequential, yet overlapping, phases: (1) hemostasis; (2) inflammatory; (3) proliferative; and (4) remodeling [21]. In the NZG-JY group, the wounds of the survived rabbits passed inflammatory phases and entered the proliferative phase, the wound edges pulling together to reduce cut defects, as shown in Figure 4. Such contraction behaviors were observed for all (4/4) surviving animals in the NZG-JY groups. However, three in four rabbits in the Quikclot group suffered severe tissue necrosis, suggesting the wound healing was still in an inflammatory phase. These observations show that NZG-JY is much better than Quikclot in accelerating the wound healing process.

### 3.3. Exothermic Reaction

**Figure 5:**
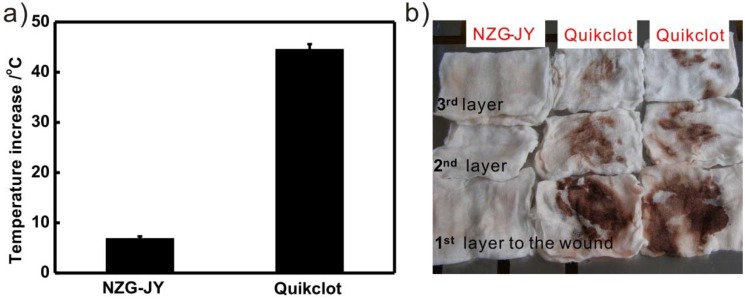
(**a**) *In vitro* exothermic experiment of NZG-JY and Quikclot. The temperature increase of water mixed with Quikclot (44.6 ± 1.0 °C) is much higher than that of NZG-JY (6.9 ± 0.4 °C); (**b**) The optical image of the first, second and third layer of the medical gauze used in the NZG-JY and Quikclot group after being washed by water. There were black spots on the gauze of the Quikclot group due to the burned tissues or blood cells, which were not observed for the NZG-JY group.

NZG-JY and Quikclot both release heat in the exothermic experiment (Figure 5a). The maximum temperature increase of NZG-JY is 6.9 ± 0.4 °C, whereas the temperature increase of the Quikclot group is 44.6 ± 1.0 °C. The temperature increase of NZG-JY decreases five-fold as compared to that of Quikclot. In the animal experiment, we can feel significant heat release when pressing on the medical gauze after applying Quikclot granules, whereas no obvious temperature change is observed for the NZG-JY group. There were also black spots on the medical gauze of the Quikclot group due to the burned tissues or blood cells, which were not observed for the NZG-JY group (Figure 5b).

The study has demonstrated that, in a lethal rabbit model of complex groin injury, hemorrhage control with NZG-JY can significantly reduce mortality from 66.7% to 21.0% (*p* = 0.005). When compared with Quikclot, the mortality was reduced from 52.6% to 21.0% (*p* = 0.044). The use of NZG-JY was also associated with a good healing property and low heat release. The hemostatic efficiency for three groups was also recorded. All animals in the NZG-JY and Quikclot group achieved hemostasis, while only 5.6% animals in the medical gauze group achieved hemostasis, which contributed to the high mortality in the group. This observation confirms that rapid hemostasis is critical for decreasing mortality in severe extremity hemorrhages.

The lethal animal model with complex groin injury was first reported by Alam *et al*. [8] The salient features of this model include a lethal, but potentially salvageabl injury, an anatomic location unsuitable for application of a tourniquet, combined arterial and venous injury with uncontrolled hemorrhage, large soft tissue and nerve injury. The above model also includes fluid resuscitation during the application of the hemostatic agents that is very difficult to be realized in battlefields and civilian victims. In our modified lethal animal model, the hemostatic agent was immediately applied without fluid resuscitation.

It is expected that the medical gauze group should have a much higher mortality than the Quikclot group (66.7% *vs*. 52.6%), since the clotting efficiency of the Quikclot group is much higher than that of the gauze group (100% *vs*. 5.6%). In a previous report, the model of extreme groin hemorrhage is designed to be 100% mortality without any treatment, and the application of standard gauze has reduced the mortality to <40% [3]. In our animal model, the insignificant mortality difference between the gauze and Quikclot suggests that there are other important factors contributing to the death of rabbits, such as tissue burning, blood loss and the psychological state of the rabbits during the testing course. In the animal experiment, it was frequently found that the anesthetized rabbits shrieked due to the tissue burning. The survival rate and material safety are two major factors that will be considered for developing new life-saving materials. Although, the model in our study lacks the measurement of blood loss and mean artery pressure. It is a highly reproducible and lethal extreme bleeding model, which replicates complex traumatic soft tissue and venous injuries associated with arterial injury occurring in the battlefield or civilian victims of vehicle accidents, street violence, wilderness accidents and construction incidents.

The wound healing property of hemostatic agents had not been previously reported. In our study, severe necrotic tissues were observed for the rabbits in the Quikclot group (3/4) a week later, suggesting that the macrophages on wound sites have not completed phagocytizing bacteria and damaged tissue, while a good recovery was found in the NZG-JY group. The entire wound healing process is a complex series of events that begins at the moment of injury and can continue for months to years. This study is a relatively first step to demonstrate that NZG-JY was effective. A follow-up study researching the cell toxicity and wound healing mechanism would be important.

Although the exothermic reaction testing was not performed *in vivo*, the *in vitro* assays in tubes with water can also represent the result *in vivo*. The hydration of the cations of the zeolite is responsible for the heat release when the zeolite is mixed with water. The much lower temperature increase in the exothermic experiment for NZG-JY is credited to the lesser Ca^2+^ content (1.93%, as show in Table 2) as compared to Quikclot, in which the Ca^2+^ content (11.43%) is five times higher [13]. The low Ca^2+^ content attenuates the heat release of NZG-JY and the resultant side effect associated with the tissue burning. Although Ca^2+^ content in NZG-JY is 1.9%, lower than its maximum concentration (3.6%), we found that the Ca^2+^ ion exchange has not led to a significant increase of Ca^2+^ concentration nor an improved procoagulant activity. This fact may be associated with its blocked microporous networks that hinder the ion-exchanging process.

**Table 2:** The element composition (wt %) and Si/Al molar ratio of NZG-JY and Quikclot.

Elements	Al	Ca	Fe	K	Mg	Na	Si/Al
NZG-JY	6.10	1.93	0.68	2.27	0.11	1.41	5.16
Quikclot	16.11	11.43	0	0	0	0.57	1

The clotting efficiency is 100% in both the NZG-JY group and Quikclot group. The hemostatic mechanism associated with the zeolite surface is still under debate. It is believed that the high hemostatic efficiency of zeolite materials is associated with their negatively charged zeolite frameworks [13]. It is a generally-accepted opinion that the blood coagulation is a contact activation process [22]. Both the NZG-JY and Quikclot are negatively charged. The negatively charged zeolite directly activated the blood zymogen factor XII to the enzyme factor XIIa, which is the first process of the intrinsic pathway of the coagulation cascade [23,24].

## 4. Conclusions

The design implemented in this study was a non-blinded, randomized animal experiment that consisted of three treatments for surgically created, reproducible, lethal extreme bleeding. The treatment groups consisted of NZG-JY, Quikclot granules and medical gauze. The hemostatic efficiency and wound healing properties of the surviving animals were compared between the NZG-JY group and Quikclot group. Both of them achieved 100% clotting efficiency. The mortality in the NZG-JY group (21.0%) is significantly less than that in the Quikclot group (52.6%) and gauze group (66.7%). More importantly, a good healing property was achieved by all surviving animals in the NZG-JY group, while three quarters of the animals in the Quikclot group had serious necrotic tissue.

In summary, the application of NZG-JY in the lethal rabbit model of groin injury significantly decreased the mortality and accelerated the wound healing process. Moreover, their low cost, easy manufacturing process and high bio-compatibility make them an excellent alternative for synthetic zeolite-type hemostatic products.

## References

[1] Achneck H.E., Sileshi B., Jamiolkowski R.M., Albala D.M., Shapiro M.L., Lawson J.H. (2010). A comprehensive review of topical hemostatic agents efficacy and recommendations for use. Ann. Surg..

[2] Galownia J., Martin J., Davis M.E. (2006). Aluminophosphate-based, microporous materials for blood clotting. Microporous Mesoporous Mater..

[3] Ward K.R., Tiba M.H., Holbert W.H., Blocher C.R., Draucker G.T., Proffitt E.K., Bowlin G.L., Ivatury R.R., Diegelmann R.F. (2007). Comparison of a new hemostatic agent to current combat hemostatic agents in a swine model of lethal extremity arterial hemorrhage. J. Trauma Inj. Infect. Crit. Care.

[4] Johansson P.I., Stensballe J., Ostrowski S.R. (2012). Current management of massive hemorrhage in trauma. Scand. J. Trauma Resusc. Emerg. Med..

[5] Dai C.L., Yuan Y., Liu C.S., Wei J., Hong H., Li X.S., Pan X.H. (2009). Degradable, antibacterial silver exchanged mesoporous silica spheres for hemorrhage control. Biomaterials.

[6] Kheirabadi B.S., Mace J.E., Terrazas I.B., Fedyk C.G., Estep J.S., Dubick M.A., Blackbourne L.H. (2010). Safety evaluation of new hemostatic agents, smectite granules, and kaolin-coated gauze in a vascular injury wound model in swine. J. Trauma Inj. Infect. Crit. Care.

[7] Cancio L.C. (2007). Comparison of a new hemostatic agent to current combat hemostatic agents in a swine model of lethal extremity arterial hemorrhage—Editorial comment. J. Trauma Inj. Infect. Crit. Care.

[8] Alam H.B., Uy G.B., Miller D., Koustova E., Hancock T., Inocencio R., Anderson D., Llorente O., Rhee P. (2003). Comparative analysis of hemostatic agents in a swine model of lethal groin injury. J. Trauma Inj. Infect. Crit. Care.

[9] Alam H.B., Burris D., DaCorta J.A., Rhee P. (2005). Hemorrhage control in the battlefield: Role of new hemostatic agents. Mil. Med..

[10] Alam H.B., Chen Z., Jaskille A., Querol R., Koustova E., Inocencio R., Conran R., Seufert A., Ariaban N., Toruno K., Rhee P. (2004). Application of a zeolite hemostatic agent achieves 100% survival in a lethal model of complex groin injury in swine. J. Trauma Inj. Infect. Crit. Care.

[11] Carraway J.W., Kent D.N., Young K., Cole A., Friedman R., Ward K.R. (2008). Comparison of a new mineral based hemostatic agent to a commercially available granular zeolite agent for hemostasis in a swine model of lethal extremity arterial hemorrhage. Resuscitation.

[12] Kheirabadi B.S., Mace J.E., Terrazas I.B., Fedyk C.G., Estep J.S., Dubick M.A., Blackbourne L.H. (2010). Safety evaluation of new hemostatic agents, smectite granules, and kaolin-coated gauze in a vascular injury wound model in swine. J. Trauma Inj. Infect. Crit. Care.

[13] Ostomel T.A., Stoimenov P.K., Holden P.A., Alam H.B., Stucky G.D. (2006). Host–guest composites for induced hemostasis and therapeutic healing in traumatic injuries. J. Thromb. Thrombolysis.

[14] Pavelic K., Hadzija M., Bedrica L., Pavelic J., Dikic I., Katic M., Kralj M., Bosnar M.H., Kapitanovic S., Poljak-Blazi M. (2001). Natural zeolite clinoptilolite: New adjuvant in anticancer therapy. J. Mol. Med..

[15] Katic M., Bosnjak B., Gall-Troselj K., Dikic I., Pavelic K. (2006). A clinoptilolite effect on cell media and the consequent effects on tumor cells *in vitro*. Front. Biosci..

[16] Rodriguez-Fuentes G., Barrios M.A., Iraizoz A., Perdomo I., Cedre B. (1997). Enterex: Anti-diarrheic drug based on purified natural clinoptilolite. Zeolites.

[17] Mumpton F.A. (1999). La roca magica: Uses of natural zeolites in agriculture and industry. Proc. Natl. Acad. Sci. USA.

[18] Rimoli M.G., Rabaioli M.R., Melisi D., Curcio A., Mondello S., Mirabelli R., Abignente E. (2008). Synthetic zeolites as a new tool for drug delivery. J. Biomed. Mater. Res. A.

[19] Acheson E.M., Kheirabadi B.S., Deguzman R., Dick E.J., Holcomb J.B. (2005). Comparison of hemorrhage control agents applied to lethal extremity arterial hemorrhages in swine. J. Trauma Inj. Infect. Crit. Care.

[20] Kilbourne M., Keledjian K., Hess J.R., Scalea T., Bochicchio G.V. (2009). Hemostatic efficacy of modified amylopectin powder in a lethal porcine model of extremity arterial injury. Ann. Emerg. Med..

[21] Ziv-Polat O., Topaz M., Brosh T., Margel S. (2010). Enhancement of incisional wound healing by thrombin conjugated iron oxide nanoparticles. Biomaterials.

[22] Vogler E.A., Siedlecki C.A. (2009). Contact activation of blood-plasma coagulation. Biomaterials.

[23] Schopka S., Schmid T., Schmid C., Lehle K. (2010). Current strategies in cardiovascular biomaterial functionalization. Materials.

[24] Baker S.E., Sawvel A.M., Zheng N., Stucky G.D. (2007). Controlling bioprocesses with inorganic surfaces: Layered clay hemostatic agents. Chem. Mater..

